# Effect of montelukast on platelet activating factor- and tachykinin induced mucus secretion in the rat

**DOI:** 10.1186/1745-6673-3-5

**Published:** 2008-02-20

**Authors:** Rene Schmidt, Petra Staats, David A Groneberg, Ulrich Wagner

**Affiliations:** 1 Department of Anesthesiology, University Medical Center, Hugstetter Strasse 55, D-79106 Freiburg, Germany; 2 Department of Medicine, Division of Pneumology, University Medical Center, Baldingerstrasse, D-35043 Marburg, Germany; 3 Institute of Occupational Medicine, Charité – Universitaetsmedizin, Free University and Humboldt University, Augustenburger Platz 1, D-13353 Berlin, Germany; 4 Department of Internal Medicine, Division of Pneumology, Klinik Loewenstein, Geißhoelzle 62, D-74245 Loewenstein, Germany

## Abstract

**Background:**

Platelet activating factor and tachykinins (substance P, neurokinin A, neurokinin B) are important mediators contributing to increased airway secretion in the context of different types of respiratory diseases including acute and chronic asthma. Leukotriene receptor antagonists are recommended as add-on therapy for this disease. The cys-leukotriene-1 receptor antagonist montelukast has been used in clinical asthma therapy during the last years. Besides its inhibitory action on bronchoconstriction, only little is known about its effects on airway secretions. Therefore, the aim of this study was to evaluate the effects of montelukast on platelet activating factor- and tachykinin induced tracheal secretory activity.

**Methods:**

The effects of montelukast on platelet activating factor- and tachykinin induced tracheal secretory activity in the rat were assessed by quantification of secreted ^35^SO_4 _labelled mucus macromolecules using the modified Ussing chamber technique.

**Results:**

Platelet activating factor potently stimulated airway secretion, which was completely inhibited by the platelet activating factor receptor antagonist WEB 2086 and montelukast. In contrast, montelukast had no effect on tachykinin induced tracheal secretory activity.

**Conclusion:**

Cys-leukotriene-1 receptor antagonism by montelukast reverses the secretagogue properties of platelet activating factor to the same degree as the specific platelet activating factor antagonist WEB 2086 but has no influence on treacheal secretion elicited by tachykinins. These results suggest a role of montelukast in the signal transduction pathway of platelet activating factor induced secretory activity of the airways and may further explain the beneficial properties of cys-leukotriene-1 receptor antagonists.

## Background

Increased production of airway mucus is one of the critical pathophysiological features of bronchial asthma, cystic fibrosis and chronic obstructive pulmonary disease (COPD) [1]. Several mediators have been identified as key players in mucus hypersecretion including acetylcholine, histamine, leukotrienes, platelet activating factor (PAF), and tachykinins [2]. The latter group belongs to a family of peptides (e.g. substance P, neurokinin A, neurokinin B) which are released from airway nerves upon stimulation [3]. We have previously demonstrated that tachykinins are potent inducers of tracheal mucus secretion in the rat [4-6]. Furthermore, others could prove the secretagogue properties of PAF in rodents, swine, and human airway tissue [7-9]. It has been postulated that PAF has the potential to generate bioactive lipids via the 5-lipoxygenase pathway, which represents a possible mechanism mediating its secretagogue properties [10-12]. In this regard, Goswami et al. could show that PAF stimulates the secretion of respiratory glycoconjugates from human airways in culture, which was totally inhibited by the experimentally used competitive leukotriene D_4 _antagonist LY 171883 [13]. The effect of clinically available cysteinyl-leukotriene-1 (cys-LT_1_) antagonists (montelukast, zafirlukast, or pranlukast) on PAF- or tachykinin induced secretory activity in the airways has never been evaluated. Therefore, it was the aim of this study to investigate the effects of montelukast on PAF- and tachykinin induced tracheal mucus secretion.

## Methods

### Reagents

Pentobarbital sodium (Nembutal^®^) for anesthesia was obtained from Sanofi (München, Germany). Sodium azide and acetylcholine were purchased from Merck (Darmstadt, Germany). Substance P, neurokinin A, and neurokinin B were from Bachem (Heidelberg, Germany). PAF was purchased from Calbiochem (Bad Soden, Germany). WEB 2086 was from Boehringer Ingelheim (Biberach, Germany). Na_2_^35^SO_4 _for radiolabelling glycoproteins was from Amersham (Braunschweig, Germany) and montelukast (MK-476) was received as a gift from Merck Frosst (Quebeck, Canada). Substance P, neurokinin A, and neurokinin B were dissolved in aqua ad injectabilia. The vehicle for PAF was ethanol. Montelukast and WEB 2086 were dissolved in dimethylsulfoxid (DMSO). Maximum concentrations of ethanol or DMSO during the experiments were 0.5%. None of the vehicles showed any significant effects on tracheal secretory activity (data not shown).

### Animals

Male Sprague-Dawley rats (Harlan Winkelmann GmbH, Borchen, Germany) with an average body weight of 436 ± 42 g were used for all experiments. The experimental protocol was approved by the local animal care and use committee, and all animals received humane care according to the criteria outlined in the *Guide for the Care and Use of Laboratory Animals *[14]. The animals were kept in a light- and temperature controlled room and had free access to water and a rat standard diet (Altromin, Lage, Germany).

### Tissue preparation

The modified Ussing chamber technique is well established for measurement of tracheal secretion and has been described in detail previously [15]. Briefly, rats were anesthetized by an intraperitoneal injection of 70 mg*kg^-1 ^body weight pentobarbital sodium. The trachea was excised through a ventral collar midline incision and median sternotomy and immediately transferred to an organ bath filled with medium M199 in Earle's balanced salt solution (Gibco, Eggenstein, Germany), equilibrated with carbogen gas (95% oxygen, 5% carbon dioxide). After removing the connective tissue, the trachea was opened along the paries membranaceus and mounted between the two halves of the modified Ussing chamber. According to the volume of the perfusion device, seven millilitres of medium M199 were added to the luminal (i.e. mucosal) and submucosal sides, respectively. The pH was adjusted to 7.41 and a constant temperature of 37°C was maintained during the whole experiment.

### Radiolabelling and measurement of airway glycoprotein secretion

50 μCi Na_2_^35^SO_4 _were added to the solution bathing at the submucosal side and allowed to equilibrate with the tissue for the duration of the experiment. After 2 h the release of bound ^35^SO_4 _to the mucosal side reaches steady state [15]. Subsequently the luminal solution was collected every 15 minutes and replaced with fresh medium. The perfusate samples from the luminal side were collected in cellulose dialysis tubing (12,000 – 14,000 Da molecular mass cut-off, Serva, Heidelberg, Germany) and dialysed against distilled water containing unlabelled Na_2_SO_4_, to remove unincorporated ^35^SO_4_, and sodium azide (10 mg*L^-1^) to prevent bacterial degradation. Dialysis was complete when the radioactive count of the dialysis fluid 3 h after the last change was the same as before dialysis. The samples were transferred to plastic vials mixed with 10 ml of szintillant (Lumagel^®^, Baker, Deventer, Netherlands) and radioactivity was measured using a liquid szintillation counter (Rackbeta LKB 1219, LKB Instruments, Graefeling, Germany). The counts of labelled macromolecules represent the secretory activity. Former studies from our lab using high-performance liquid chromatography (HPLC) and autoradiography identified these labelled macromolecules as airway secretory glycoproteins from the submucosal glands, which were not digested by chondroitinase ABC. Thus, these macromolecules are true glycoproteins.

### Experimental design

After two hours of incubation, samples were collected every 15 minutes. The average of two samples before pharmacological intervention represented the basal secretion rate (= 100%). Drugs were applied to the mucosal side and collections were taken 15 minutes later. Between each application, at least four samples were collected to allow the system to recover and reach a basal secretion again. In order to test the viability of the system, each experiment was finished with a stimulation of acetylcholine (1 μM), an established secretagogue for this system.

### Data analysis

Data are expressed in percent of basal secretion ± SEM. Statistical analysis was performed with Student's *t*-test for paired samples. Experiments with five animals per group were performed for each experimental protocol. Data were considered significant when P < 0.05. Statistical analysis was performed using the Sigma Stat software package (Jandel Scientific, San Rafael, CA).

## Results

### Effect of WEB 2086 on PAF induced tracheal secretory activity

**Figure 1:**
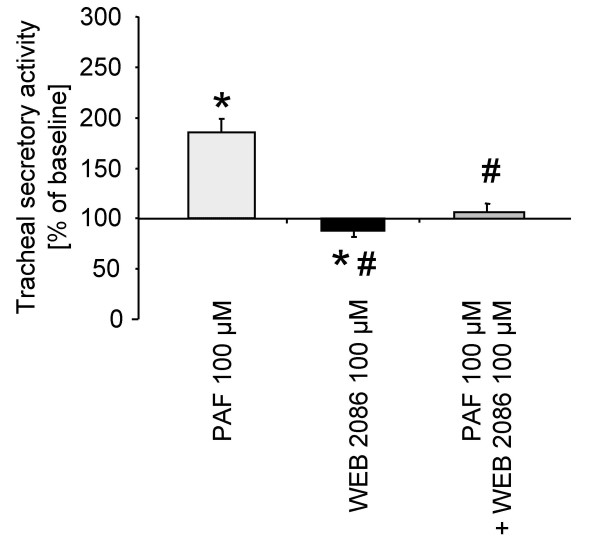
**Effects of WEB 2086 on platelet activating factor (PAF) induced tracheal secretory activity in the rat.** Data are expressed as mean ± SEM for n = 5 animals per group. *P < 0.05 versus respective baseline secretion values (within each group); ^#^P < 0.05 versus PAF.

The effect of the PAF receptor antagonist WEB 2086 on PAF induced tracheal secretory activity is depicted in figure 1. PAF (100 μM) stimulates secretion significantly to levels up to 185 ± 10% of baseline. Application of WEB 2086 (100 μM) led to a moderate suppression of baseline secretion (85 ± 5%). Co-administration of PAF (100 μM) and WEB 2086 (100 μM) abolished the increase of secretion observed under PAF application alone (105 ± 10% of baseline).

### Effect of montelukast on PAF induced tracheal secretory activity

**Figure 2:**
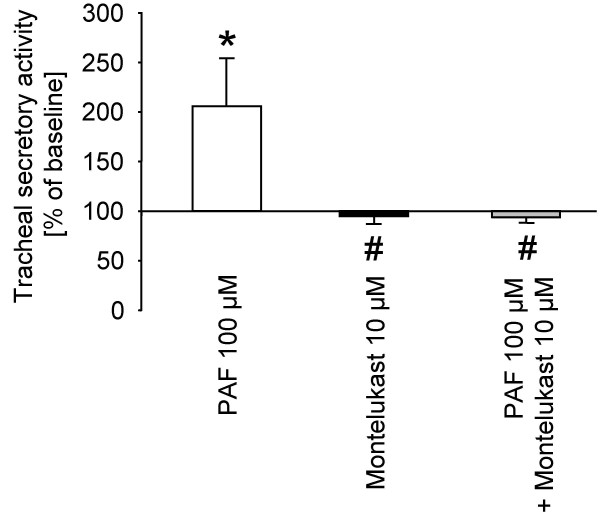
E**ffects of montelukast on platelet activating factor (PAF) induced tracheal secretory activity in the rat.** Data are expressed as mean ± SEM for n = 5 animals per group. *P < 0.05 versus respective baseline secretion values (within each group); ^#^P < 0.05 versus PAF.

Figure 2 shows the influence of the cys-LT_1 _receptor antagonist montelukast on PAF induced tracheal secretory activity. PAF application (100 μM) led to an increase of mucus secretion up to 205 ± 49% of baseline levels. The addition of montelukast (10 μM) to the culture medium had no significant effect on the secretion levels (95 ± 6%). Combination of PAF (100 μM) and montelukast (10 μM) completely blocked the secretagogue effect observed under PAF application alone (94 ± 5%).

### Effect of montelukast on substance P, neurokinin A, and neurokinin B induced tracheal secretory activity

**Figure 3:**
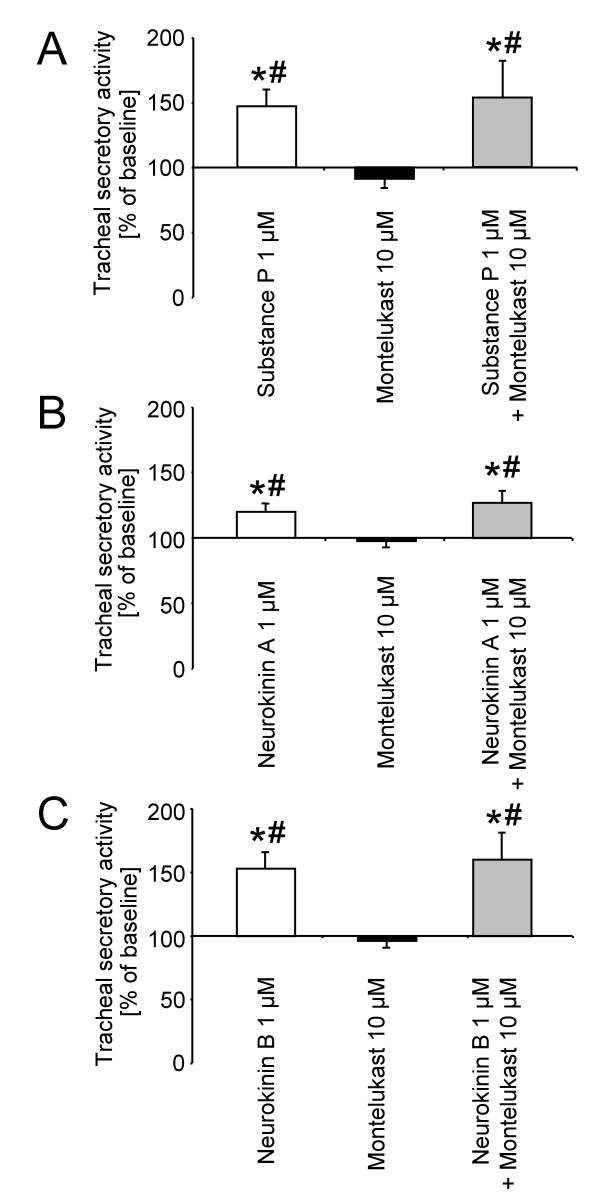
**Effects of montelukast on tachykinin (substance P (A), neurokinin A (B), neurokinin B (C)) induced tracheal mucus secretion in the rat.** Data are expressed as mean ± SEM for n = 5 animals per group. *P < 0.05 versus respective baseline secretion values (within each group); ^#^P < 0.05 versus montelukast.

As shown in figure 3A, substance P (1 μM) stimulated tracheal secretory activity significantly. Montelukast administration (10 μM) alone exerted no effect on baseline secretion (91 ± 3%) and had no modulating capacity on substance P induced mucus secretion (substance P: 147 ± 14%; substance P + montelukast: 153 ± 28%). Figure 3B depicts the effect of montelukast on neurokinin A induced tracheal secretory activity. Neurokinin A (1 μM) increased secretion significantly (120 ± 7%). Montelukast alone had no effect on baseline secretion (98 ± 5%) and could not influence the neurokinin A induced increase of tracheal secretion (neurokinin A + montelukast: 127 ± 9%). The effect of montelukast on neurokinin B induced tracheal mucus secretion is presented in figure 3C. Neurokinin B (1 μM) stimulated mucus secretion (153 ± 12%). Montelukast alone did not modulate the basal secretion rate and had no influence on neurokinin B induced mucus secretion when given in combination (montelukast: 98 ± 5%; neurokinin B + montelukast: 160 ± 21%).

## Discussion

The aim of the present study was to characterize the effects of the clinically used cys-LT_1 _receptor antagonist montelukast on PAF- and tachykinin induced tracheal secretory activity in the rat. Our results could demonstrate that PAF potently stimulates tracheal mucus secretion. This could be completely blocked by administration of the selective PAF receptor antagonist WEB 2086 as well as montelukast. In addition, we could show that the tachykinins substance P, neurokinin A, and neurokinin B also significantly increased tracheal mucus secretion. In contrast to the inhibition of PAF induced secretion, montelukast did not modulate tachykinin stimulated secretory activity.

Recently, we demonstrated that the cys-LT_1_-receptor antagonist zafirlukast is a potent stimulator of tracheal secretion in the rat [16]. In contrast, montelukast has much lower potency and does not exert secretagogue effects until concentrations of 100 μM are reached. Therefore, we used 10 μM montelukast in the present study to evaluate the effects of this cys-LT_1 _receptor antagonist on PAF and tachykinin stimulated tracheal secretory activity in the rat.

The naturally occurring phospholipid mediator PAF (1-O-alkyl-2-acetyl-*sn*-glycero-3-phosphocholine) is produced by a variety of inflammatory cells including neutrophils, alveolar macrophages, mast cells, eosinophils, and others. PAF originates from cleavage of membrane phospholipids by phospholipase A_2 _yielding lyso-PAF, which is further acetylated to form biologically active PAF. Its degradation to the inactive lyso-PAF is catalysed by a PAF-specific acetylhydrolase, which is abundantly present in plasma and intracellularly in several inflammatory cells [17]. PAF supports the pathogenesis of many inflammatory reactions, including airway inflammation. Besides bronchoconstriction, microvascular leakage, recruitment and activation of eosinophils and airway hyperresponsiveness, PAF is seriously involved in mucus hypersecretion which is a critical feature of the inflammatory process and occurs during asthma, chronic obstructive airway disease, or pneumonia [18]. PAF has been shown to serve as a powerful mucus secretagogue in the airways of animals and humans [13,19]. The mechanism of PAF induced airway hypersecretion has been extensively studied during the last years. It could be demonstrated that the PAF mediated effect does not depend on a cholinergic mechanism or the generation of histamine. In contrast, accumulating evidence supports the notion that the pulmonary effects of PAF could be mediated by the secondary release of leukotrienes [18]. It is now widely accepted that a significant amount of peptidoleukotrienes are generated in response to a PAF challenge and that these products of the arachidonic acid metabolism are at least in part responsible for the proposed PAF mediated effects [20,21]. In addition, it could be shown that inhibition of the arachidonic acid pathway by administration of dexamethasone or inhibitors of the lipoxygenase or cyclooxygenase pathway completely blocked the secretagogue properties of PAF [7,13,21]. Furthermore, the experimentally used leukotriene receptor antagonist LY 171883 totally inhibited PAF-induced secretion of respiratory glycoconjugates from human airways in culture, indicating a critical role for leukotrienes in PAF induced hypersecretion [13]. The results of the present study confirm these data and add new information concerning the clinically used cys-LT_1 _receptor antagonist montelukast. While the administration of montelukast alone had no effect on tracheal secretory activity, it completely inhibited PAF stimulated airway secretion in our setting. Regarding this effect, montelukast was as effective as the specific PAF receptor antagonist WEB 2086.

In addition, we could confirm earlier studies from our group indicating the secretagogue properties of the tachykinins substance P, neurokinin A, and neurokinin B in the same model. Nevertheless and unlike our previous results, neurokinin B exerted more potent secretagogue effects than neurokinin A in the present experimental series. Furthermore, mucus secretion in response to stimulation with the tachykinins was slightly lower when comparing earlier studies from our group with the results of the present investigation. It has been shown that the secretory activity of the airways could be influenced by the circadian rhythm, which could be one explanation for these differences. Moreover, the Ussing chamber position on the tracheal surface critically affects the amount of secreted mucus macromolecules and variations in that regard could also not be excluded. Crimi and colleagues have shown in human patients that montelukast abolishes the bronchoconstrictor airway response to neurokinin A, lending support to the hypothesis that tachykinins might elicit bronchoconstriction indirectly through the release of cys-LTs [22]. In sharp contrast to the abovementioned action in the context of bronchoconstriction, montelukast did not modulate neither substance P nor neurokinin A or neurokinin B stimulated tracheal secretory activity in our setting.

## Conclusion

In conclusion, our data show that the clinically used cys-LT_1 _receptor antagonist montelukast inhibits PAF induced tracheal secretory activity to the same degree as the specific PAF receptor antagonist WEB 2086. No modulating effect could be demonstrated after montelukast administration when airway secretion was stimulated by tachykinins. These findings may contribute to the beneficial effect of montelukast in the treatment of bronchial asthma.

## Competing interests

The author(s) declare that they have no competing interests.

## Authors' contributions

RS, PS, DAG and UW have been involved in the design and conduct of the study. Also they have participated in drafting the article or revising it critically for important intellectual content. They have all given approval of the study to be published.
